# Freeform micropatterning of living cells into cell culture medium using direct inkjet printing

**DOI:** 10.1038/s41598-017-14726-w

**Published:** 2017-11-03

**Authors:** Ju An Park, Sejeong Yoon, Jimin Kwon, Hesung Now, Young Kwon Kim, Woo-Jong Kim, Joo-Yeon Yoo, Sungjune Jung

**Affiliations:** 1 Department of Creative IT Engineering, Pohang University of Science and Technology (POSTECH), 77 Cheongam ro, Nam-gu, Pohang, Kyungbuk 37673 Republic of Korea; 2 School of Interdisciplinary Bioscience and Bioengineering, Pohang University of Science and Technology (POSTECH), 77 Cheongam ro, Nam-gu, Pohang, Kyungbuk 37673 Republic of Korea; 3 Department of Life Sciences, Pohang University of Science and Technology (POSTECH), 77 Cheongam ro, Nam-gu, Pohang, Kyungbuk 37673 Republic of Korea; 4 Department of Mechanical Engineering, Pohang University of Science and Technology (POSTECH), 77 Cheongam ro, Nam-gu, Pohang, Kyungbuk 37673 Republic of Korea

## Abstract

Microfabrication methods have widely been used to control the local cellular environment on a micron scale. However, accurately mimicking the complexity of the *in vivo* tissue architecture while maintaining the freedom of form and design is still a challenge when co-culturing multiple types of cells on the same substrate. For the first time, we present a drop-on-demand inkjet printing method to directly pattern living cells into a cell-friendly liquid environment. High-resolution control of cell location is achieved by precisely optimizing printing parameters with high-speed imaging of cell jetting and impacting behaviors. We demonstrated the capabilities of the direct cell printing method by co-printing different cells into various designs, including complex gradient arrangements. Finally, we applied this technique to investigate the influence of the heterogeneity and geometry of the cell population on the infectivity of seasonal H1N1 influenza virus (PR8) by generating A549 and HeLa cells printed in checkboard patterns of different sizes in a medium-filled culture dish. Direct inkjet cell patterning can be a powerful and versatile tool for both fundamental biology and applied biotechnology.

## Introduction

Similar to how painters capture and depict the real world on a canvas, tissue engineers construct and rebuild human bodies *in vitro*, often in a plastic dish^1,2^. Living cells have been micropatterned into predetermined designs *in vitro* to mimic the complex microenvironment of our bodies. Representative cell micropatterning methods include photolithography^3–5^, soft lithography^6,7^, and microfluidics^8–11^. Photolithography techniques use successive UV treatment steps and toxic solvents to develop sub-micrometer-scale patterns on a surface that allow cells and proteins to adhere. Soft lithography techniques use bio-friendly soft elastomeric stamps to fabricate micrometer- to nanometer-scale patterns, with multiple mold fabricating steps required to generate the stamps. Microfluidic channels offer 3D dynamic flows in channels to create patterns and enable the simultaneous patterning of multiple materials; the complexity of the patterns is closely related to the number of channels, which are formed by an intricate fabrication process. These conventional micropatterning methods have enabled closer and deeper investigations of cellular behaviors, biological phenomena, and interactions between a physiological environment and cells than traditional 2D cell culture. However, these methods involve complicated and time-consuming fabrication steps and require substantial technical knowledge or experience with surface engineering using harmful organic materials. Another challenge is the correct positioning of multiple cell types on the same substrate using various design patterns.

Recently, bioprinting has become a promising micropatterning technology that adopts bottom-up and scaffold-free fabrication styles. Bioprinting may meet the requirements of a simple and flexible yet a versatile fabrication technology. Sub-types of bioprinting technology include microextrusion^12–15^, inkjet^16–24^, laser^25–28^, valve-based^29,30^, electrohydrodynamic^31,32^ and acoustic bioprinting^33,34^. Among these methods, inkjet printing offers many advantages, including high resolution and precision, a fast printing speed, a low cost, and compatibility with many biomaterials^35–37^. With its drop-on-demand drop generation mechanism, this printing technique also has the potential to introduce concentration gradients of cells and biomaterials by altering drop densities or sizes. However, the majority of bioprinting methods, including inkjet cell printing, utilize supporting hydrogels either as an ink supplement or as a substrate to fix and sustain the positions of the printed cells without dehydration and cellular damage. The use of chemical compounds may produce potentially cytotoxic remnants that induce protein denaturation.

Here, we describe a highly accurate direct inkjet printing system and process for directly patterning living cells into a liquid medium-filled cell culture dish. Printing conditions, such as stage speed, stand-off distance, and the medium volume in the cell culture dish, were systematically optimized to ensure the high resolution and reliability of the process and to establish fine and complex cell patterns in the cell culture medium. We used high-speed stroboscopic and cinematographic imaging techniques to monitor cell jetting and impact behaviors. Our method does not involve any surface engineering (e.g., UV treatment), harmful chemical treatments (e.g., photoresist treatment), specialized substrates (e.g., silicon wafers), supporting hydrogels (e.g., collagen) other than a cell culture medium, or the fabrication of masks, molds, and stamps. After examining cell viability and proliferation, we report the ability to create co-culture systems by patterning NIH3T3 and HEK293A cells into various designs, such as straight and curved lines, solid areas, and complex gradient patterns. As a case study, we produced A549 and HeLa cells printed into checkerboard patterns of different sizes to determine how the heterogeneity and the geometry of cell populations affect the infectivity of a seasonal H1N1 influenza virus (PR8).

## Results

### Direct inkjet-based cell printing system and process

**Figure 1:**
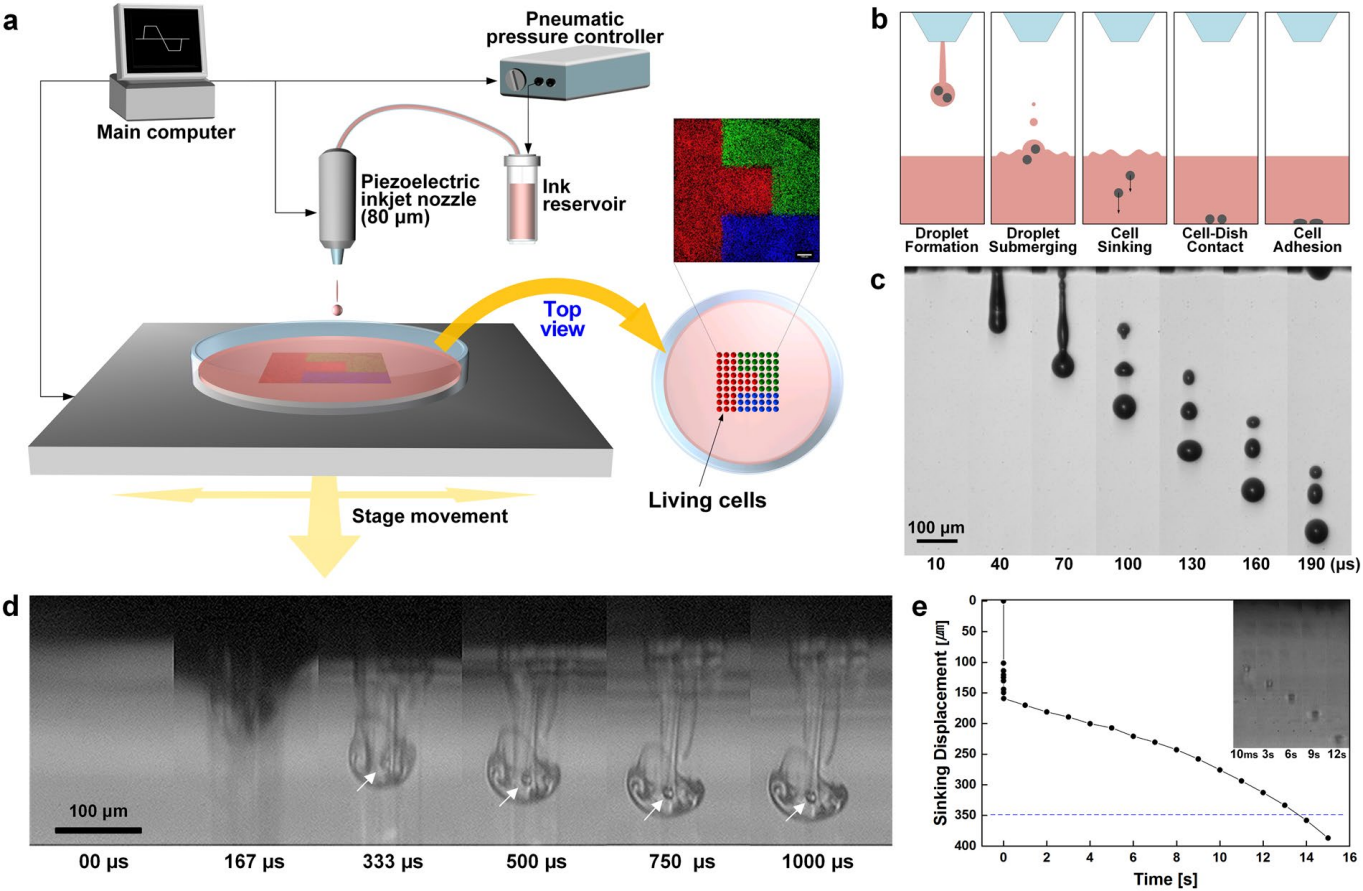
Direct inkjet cell printing system and process. (**a**) Schematic of the apparatus used for direct inkjet cell patterning with a 3-axis controllable piezoelectric inkjet printing system. Cell-laden ink is printed from an ink reservoir through an 80-µm piezoelectric inkjet nozzle onto a liquid-filled cell culture dish. A computer controls the stand-off distance by adjusting the movement of the nozzle in the z direction and pattern design by adjusting the movement of the stage in the x and y directions. (**b**) Schematic illustration of the process used to pattern cells in a liquid-filled plate. A cell-laden drop is ejected from an inkjet nozzle, submerged in liquid substrate, and then cells in the drop sink, contact, and adhere to the bottom of the plate, where they remain on the printed position. (**c**) High-speed stroboscopic images of the jetting of a cell-laden drop were obtained with a CCD camera and a spark flash light at 30-µs intervals. (**d**) High-speed cinematographic images of sinking cells after a drop was impinged into a liquid-filled substrate. Images of a drop submerging and the cell sinking in a liquid medium were obtained at a rate of 12,000 frames per second using high-speed imaging. For visualization, the cell culture medium bath was replaced with 99% ethanol. White arrows indicate living cells sinking under the liquid pool after drop impact. (**e**) Tracking the displacement of sinking cells at different time intervals. The displacement of sinking cells was measured 10 times. The blue dashed line indicates the optimized depth (~350 µm) of the cell culture medium used to print cell patterns. Images shown in the upper right panel are representative images of sinking cells captured at each time point.

We inkjet-printed cell-laden ink onto predestined locations of a cell culture dish filled with cell culture medium (Fig. 1a). Complex cell patterns were fabricated using a computer-controlled printing system with a motorized x-y stage and a z-axis elevating nozzle. As ink, living cells were suspended in cell culture medium at a density of 6 × 10^6^ cells mL^−1^. The shear viscosity of the cell-laden ink was evaluated before printing (Supplementary Fig. 1). Cells were printed from an ink reservoir through an 80-μm-diameter nozzle and plunged into a pool of liquid medium to be patterned. Living cells were aligned onto predetermined patterns through multiple steps (Fig. 1b). First, cell-laden drops were ejected from the tip of an inkjet nozzle using an applied piezoelectric signal input. The stages of drop formation were captured in single-flash images (Fig. 1c). Next, the ejected cells were plunged into medium in a culture dish (Fig. 1d and Supplementary Video). A vortex ring was formed when the drop coalesced with the liquid medium. The vortex quickly propagated into the liquid for ~1 ms and became wider as it slowed down. The initial penetration depth was ~167 µm, and then the cells slowly dispersed into the liquid at a speed of ~14.2 µm s^−1^ until they reached the bottom of the dish (Fig. 1e). Over time, cells adhered to the bottom of the dish in the printed patterns.

### Effects of printing parameters on accuracy

**Figure 2:**
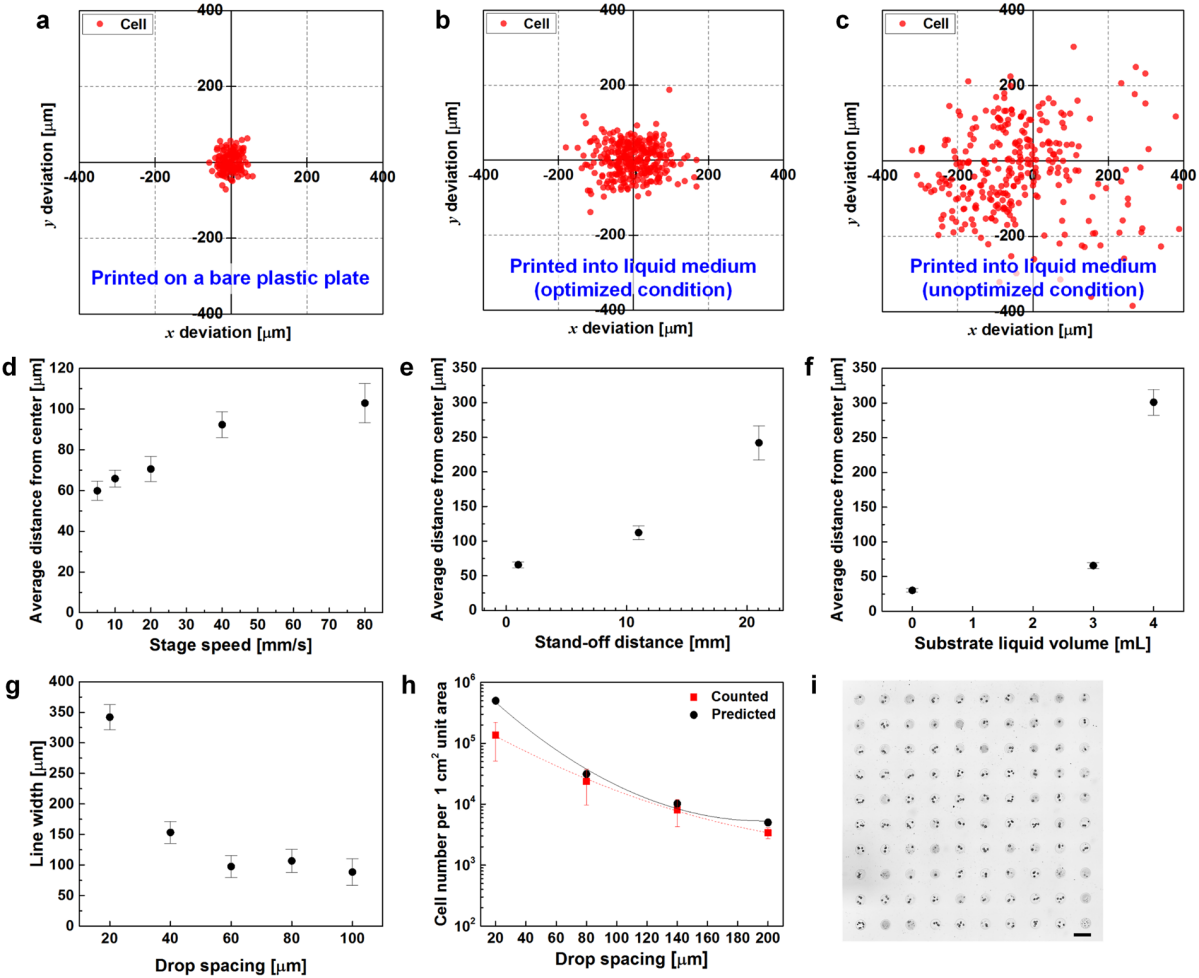
Position accuracy and control of the density of cells printed on liquid substrates using direct inkjet printing. (**a**) Positional deviations of cells printed on a bare polystyrene cell culture dish without liquid. Red dots represent the locations of the cells after sinking. (**b** and **c**) Cells printed onto a cell culture dish containing DMEM under optimized and unoptimized conditions, respectively. (**d**–**f**) Average distance of cell position from the center vs. stage speed, stand-off distance, and liquid volume in dishes, respectively. Error bars indicate the 95% CIs. (**g**) Line width vs. drop spacing. Error bars indicate the 95% CIs. (**h**) Density of printed cells (cells cm^−2^) at different drop spacing intervals. Differences were not significant according to the two-way ANOVA followed by Tukey’s post hoc test, p > 0.05. Error bars indicate the s.e. (**i**) Representative images of printed cells in a drop. Cell-laden drops were printed as 10 × 10 array with 0.4-mm spacing. Scale bar: 200 μm. Average number of cells/drop: ~2.

We investigated the correlation between printing parameters and positional accuracy of the printed cells. NIH3T3 cells were printed on the bare surface of a cell culture dish with a positional deviation of ± 30 µm from the designated location (Fig. 2a). Once cells were printed into a liquid medium-filled dish, the positional deviation of cells increased compared with that of cells printed onto the plastic surface (Fig. 2b and c). Coordinates of printed cells in the medium were extracted by image processing and the distance between cell positions and their averaged center positions were calculated to define cell printing resolution (Supplementary Fig. 2). The optimization of printing conditions yielded an error of ± 66 µm, representing a significant improvement from the unoptimized method. We have systemically investigated the effects of printing parameters, such as stage speed, stand-off distance, and the volume of medium in a cell culture dish, on the reliability and resolution of cell patterning. Increasing the speed of the moving stage from 5 to 80 mm s^−1^ and the stand-off distance, which is the gap between the inkjet nozzle and the substrate, from 1 to 21 mm adversely affected the accuracy of the printed cell position (Fig. 2d and e). The analysis of the resolution in dishes containing 3 mL of medium showed a minimal difference from the results obtained with the bare dishes, and volumes exceeding 3 mL showed larger deviations (Fig. 2f). The minimum volume of medium required to minimize unwanted dehydration was 3 mL.

Drop spacing, which is the gap between two consecutive drops, was controlled by varying the speed of the motion stage or the frequency of drop generation. Linewidth depended on the drop spacing. Cell-laden drops with a narrow drop spacing of less than 60 µm formed a thicker linewidth, which was not changed for greater drop spacing distances (Fig. 2g). The number of printed cells in a unit area was controlled by varying the drop spacing. As shown in Fig. 2h, the cell density decreased from ~10^5^ to ~10^3^ cells cm^−2^ after the drop spacing was adjusted from 20 to 200 µm. Using a cell-ink concentration of 6 × 10^6^ cells mL^−1^, an average of approximately 2 cells were deposited in a printed drop using our printing process (Fig. 2i and Supplementary Fig. 3). The number of cells per 1 cm^2^ unit area was manually counted from captured images and compared to the calculated value of the average number of cells, which exhibited a good agreement. Printed cell density curves closely followed the calculated curves, with F = 1.08482, and p > 0.05, as calculated using two-way ANOVA.

### Cell viability and proliferation test

**Figure 3:**
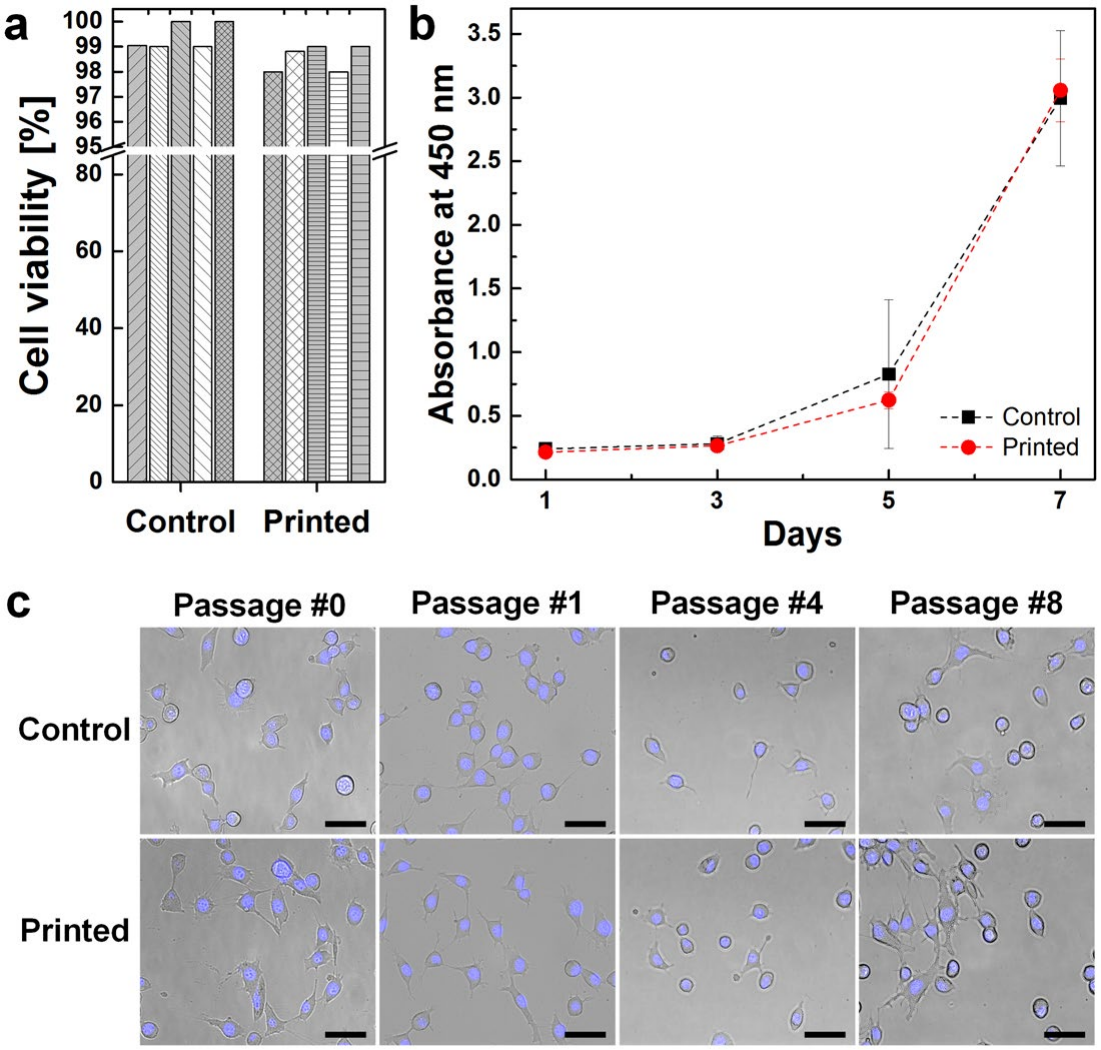
Post-printing cell viability tests. (**a**) The LIVE/DEAD assay was used to measure cell survival immediately after printing. Each bar indicates an individual assay (*n* = 5). (**b**) The proliferation rate was measured every other day for 7 days. No significant differences were observed using the two-way ANOVA followed by Tukey’s post hoc test, p > 0.05; error bars indicate the s.d. (*n* = 5). (**c**) Long-term cell morphology was monitored until passage 8. Cells from both the printed and control groups maintained their original morphologies. Nuclei were stained with Hoechst 33342 dye. Scale bar: 50 μm.

We next examined cell viability and proliferation using 3 different assays: instant measurements with the LIVE/DEAD assay kit immediately after cell printing, tracking the cell proliferation rate for one week, and long-term inspections of cellular morphology in maintenance cultures for 3 weeks. The results of the LIVE/DEAD assay showed negligible disparity in instant viability between the printed group and the pipetted control group (Fig. 3a). Viabilities of the printed group and the control group were 98.6% and 99.0%, respectively. Over one week, cells from the printed group closely followed the growth curve of the pipetted control group, with F = 0.06485. No significant differences were observed between groups (p > 0.05) in the two-way ANOVA (Fig. 3b). Moreover, the proliferation observed on day 7 was higher for printed cells than the control group, due to the proliferation of the printed cells. In images of cell acquired after printing, cells maintained their morphology and remained alive for 3 weeks, representing 8 passages (Fig. 3c). No obvious signs of chromosomal fragmentation or cell damage observed.

### Fabrication of cell patterns

**Figure 4:**
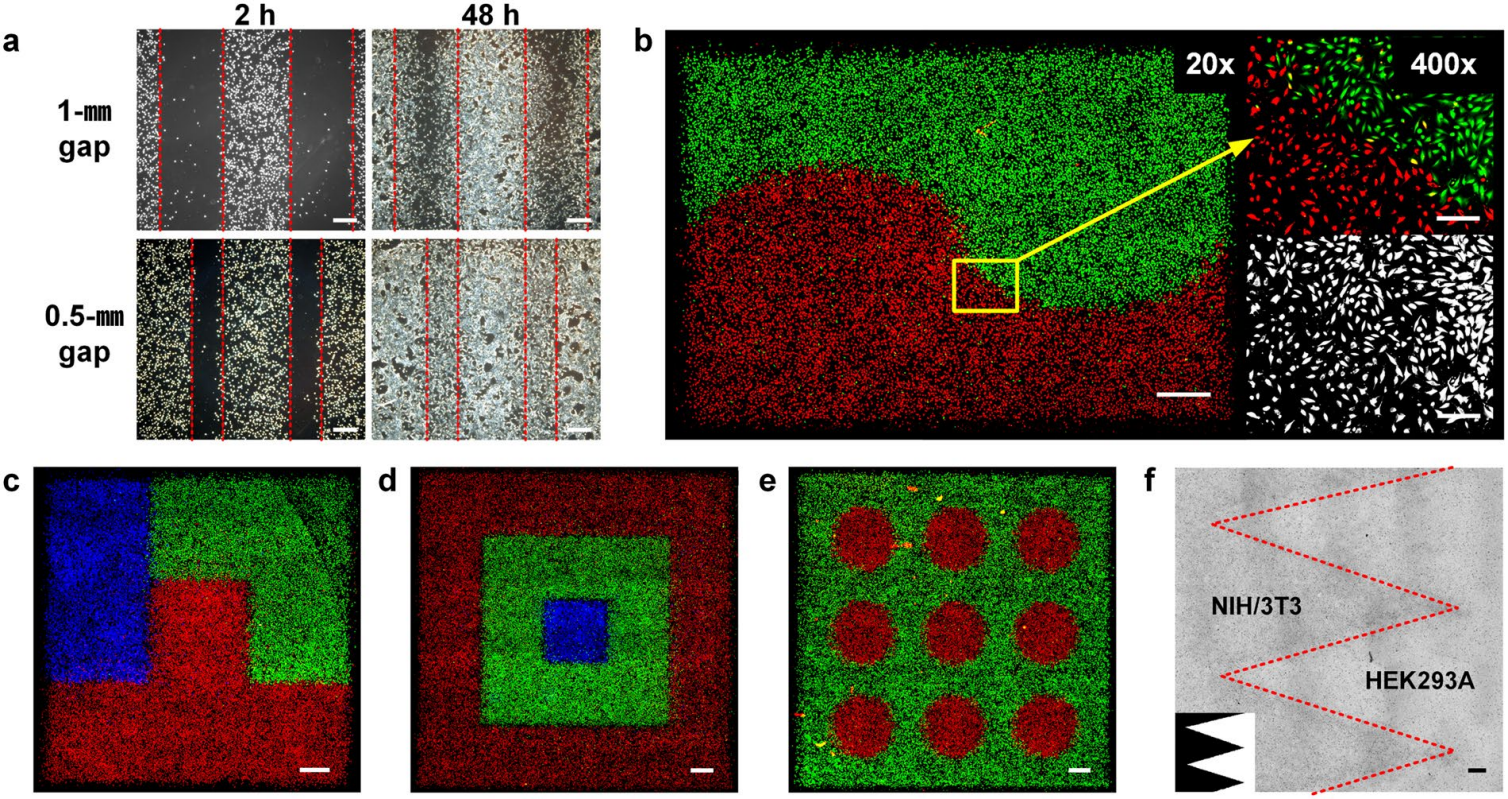
Demonstration of various cell patterns that were directly printed into culture medium. (**a**) Printed cell migration assay model with 1-mm and 0.5-mm gaps. Images were captured at 2 hours and 48 hours after printing. Scale bar: 500 µm. (**b–e**) Fluorescent images of red-, green-, and blue-labeled cell patterns. Cells were pre-labeled and printed in patterns. Scale bar: 1 mm. (**f**) Printed zigzag pattern using a heterotypic co-culture model of NIH3T3 and HEK293A cells. Scale bar: 1 mm.

We used the direct inkjet printing process to pattern various designs of NIH3T3 and HEK293A cells. First, Fig. 4a shows migration assay models printed on a cell culture dish that were used to track and quantify cellular behaviors in wound healing or drug response assays. NIH3T3 cells were patterned with a controlled density, leaving 0.5- and 1-mm spaces. Images were captured daily to show gap closure induced by cell proliferation and migration over time. Next, NIH3T3 cells were labeled with 3 different fluorescent dyes, red, green, and blue (RGB), and patterned to demonstrate the possibility of heterotypic cell patterning (Fig. 4b–e). Cells were printed into sufficiently fine patterns to show curved designs and the distinction between edge boundaries of different colored groups. After validating the possibility of fabricating a heterotypic co-culture model using RGB-labeled cells, a genuine heterotypic co-culture model was printed using different cell lines (Fig. 4f). NIH3T3 mouse fibroblasts and HEK293A human kidney cells were co-patterned into Dulbecco’s Modified Eagle’s Medium (DMEM) with a zigzag interface. The boundaries of the zigzag pattern were clearly observed, as regions containing NIH3T3 cells appeared to be noticeably darker than regions containing HEK293A cells.

**Figure 5:**
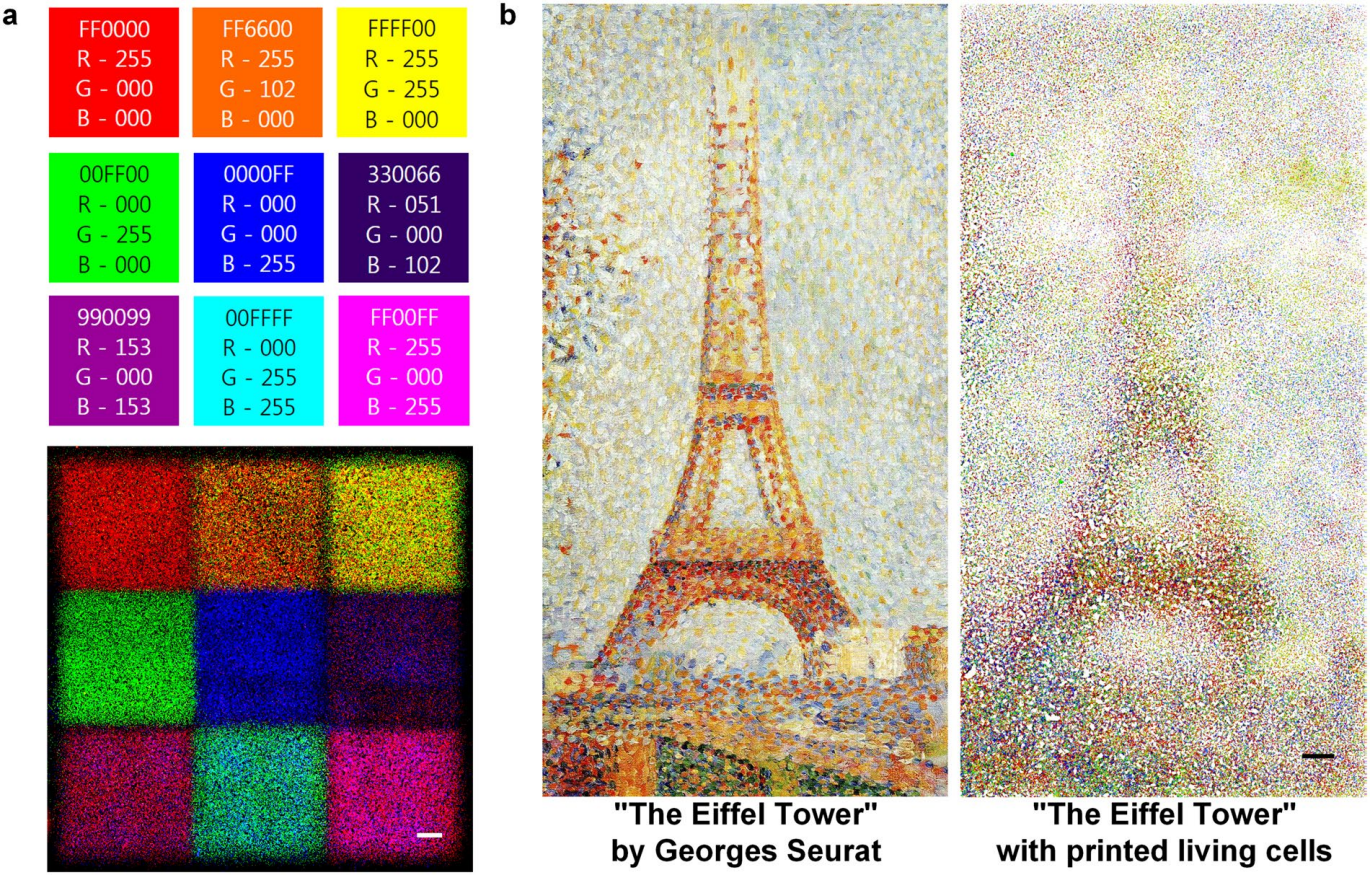
Density-controlled 2D cell patterns using direct inkjet cell printing. (**a**) Nine-color chart printed with cells labeled with red, green, blue (RGB) dyes. The nine colors were generated by controlling the combination and density of RGB-labeled cells. Scale bar: 1 mm. (**b**) “The Eiffel Tower” by Georges Seurat was patterned with cells labeled with 3 colored dyes. The image of the original painting is from The Fine Arts Museum of San Francisco. Scale bar: 1 mm.

In addition to the high-resolution patterning, another advantage of direct cell printing is its capability to introduce concentration gradients of cells by varying the drop densities. We patterned a color chart with 9 major colors consisting of the 7 rainbow colors, cyan and magenta by simply printing 3 RGB-labeled cell inks to show the ability to pattern cells in concentration gradients (Fig. 5a). Cells were patterned in 9 sectioned squares according to preset RGB color ratios obtained from the desired 9 colors. For example, the orange color at the center of the first row was pigmented by printing 3 RGB-labeled cells at a 5:2:0 ratio, respectively. This strategy exemplifies the control over cell density and patterning in a single dish in one step. After validating variegated color expression with only 3 colored cells, we attempted to mimic a masterpiece, “The Eiffel Tower” by Georges Seurat. We printed RGB-labeled cells in a medium to pattern the painting by employing tiny juxtaposed colored dots, similar to the method used by this French Post-Impressionist painter (Fig. 5b). The effective control of the cell density gradient and the high resolution of our inkjet printing method created a gradual and serial assortment of cells in “The Eiffel Tower” printing. Local control over the cell density enabled us to visualize and imitate the original features in a similar impression.

### Viral infection

**Figure 6:**
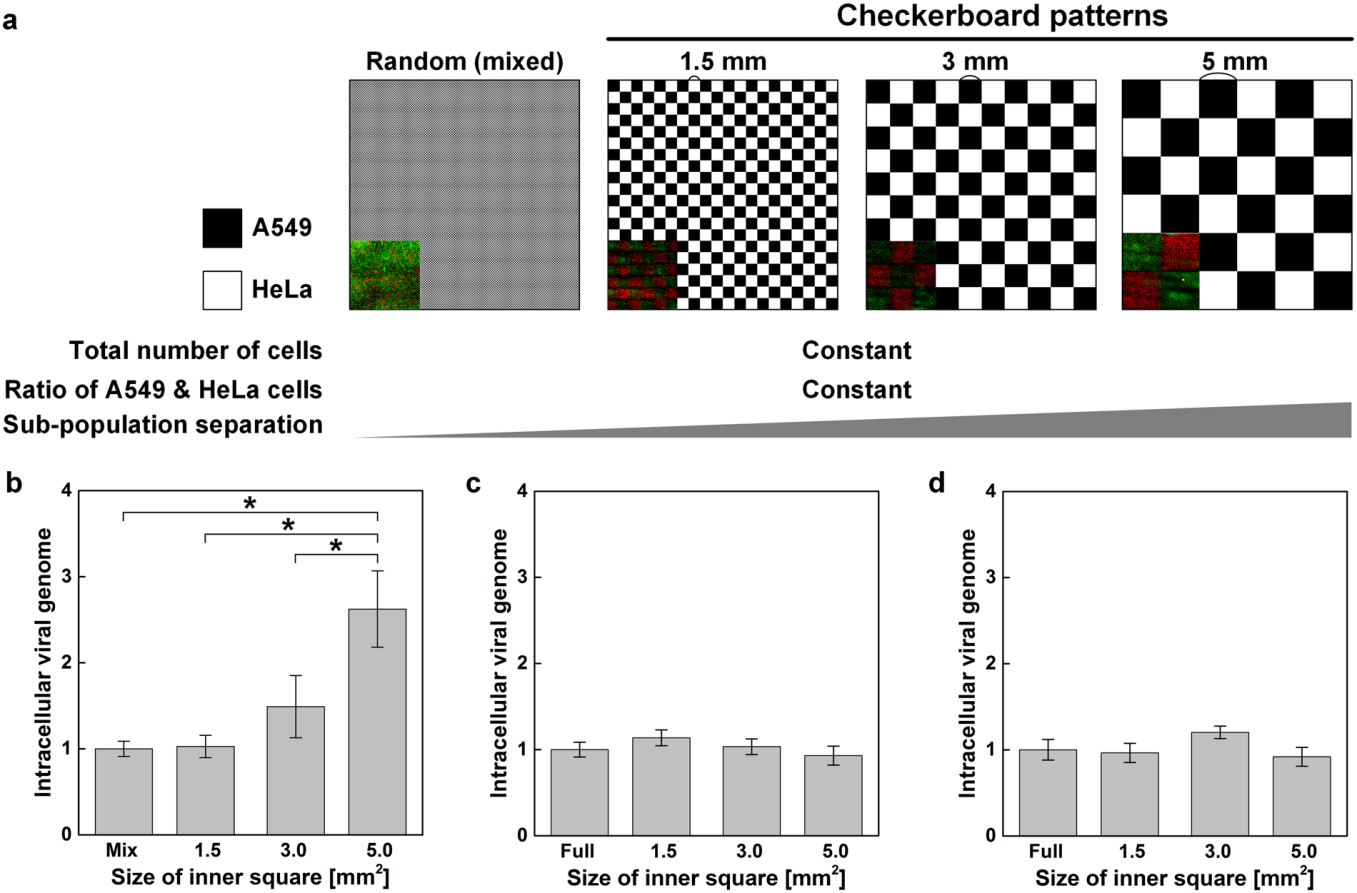
Induction of differential susceptibilities to viral infection through the geometric separation of heterogeneous cell populations. (**a**) Four types of checkerboard designs with inner squares of different sizes were generated by printing cell patterns using direct inkjet printing. The inset images are the real cell patterns of red HeLa cells and green A549 cells. (**b**) After patterning heterotypic A549 and HeLa cells according to the designs shown in (**a**), cells were incubated for 48 hours and infected with influenza virus (10 TCID50 mL^−1^) for 24 hours. Cells were harvested and the amount of the influenza HA viral RNA (normalized to GAPDH levels) was measured using quantitative real-time PCR. Means and standard errors were obtained from three independent experiments and analyzed by one-way ANOVA followed by the Newman-Keuls post hoc test, *p < 0.05. (**c**) Total viral genome content measurement from A549 cell-only patterns and (**d**) HeLa cell-only patterns.

As a case study, we produced a viral infection model with two heterotypic cells to study the influence of the heterogeneity and geometry of the cell population on the infectivity of a seasonal H1N1 influenza virus (PR8). Using the direct inkjet printing process, A549 and HeLa cells were printed into a medium-filled cell culture dish in checkerboard patterns of different sizes, where the width of the single squares was 1.5, 3, or 5 mm and the width of the total pattern size was 30 mm (Fig. 6a). A549 and HeLa cells are known to possess a high and a low susceptibility to the influenza virus, respectively^38,39^. As a control group, the cell inks were homogeneously mixed with 1:1 ratio before printing and patterned into a single large square. Differences in the geometric heterogeneity of cell populations were established using the square patterns while maintaining constant overall cell densities and ratios between the two cell types.

After infection with the H1N1 virus, the amount of total viral genome present in the entire population was monitored by measuring the overall infectivity of each heterogeneous population (Fig. 6b). Remarkably, overall infectivity or the susceptibility of the population to virus infection changed significantly with the size of the inner square. The control group with a randomly mixed sub-population showed similar intracellular viral genome contents to the 1.5-mm-sized checkerboard group. However, as the inner square size increased from 1.5 mm to 3.0 and 5.0 mm, the total intracellular viral genome contents also increased. Heterogeneous cell populations with checkerboard patterns comprising larger areas were more susceptible to infection than were populations with smaller patterns. Contrary to the multiple cell model, the viral infectivity of either A549 or HeLa cell pattern was not affected by the size of the pattern (Fig. 6c and d). Single-type cells were printed into checkerboard patterns with 1.5, 3, 5 and fully filled 30 mm sized inner squares leaving the areas between the cell-filled squares blank. Based on this result, we suggested that the viral susceptibility of the entire population composed with two subpopulations was not explained by the arithmetic summation of the susceptibility of individual cells, but influenced by the geometric heterogeneity.

## Discussion

Different patterning strategies have been utilized to obtain a fine level of control over the cellular position, organization, interactions in in-depth explorations of the microenvironment in which cells are embedded. Current patterning techniques, such as photolithography, soft lithography, and microfluidics, have enabled the fabrication of millimeter- to nanometer-scale patterns of cells and proteins to help researchers understand biological and physiological phenomena occurring in our bodies using an *in vitro* system. However, these micro and nanotechnologies for cell patterning are generally limited in their capability to mimic complex and large tissues in a cell-friendly environment^2^. Drop-on-demand inkjet printing can address these limitations by directly patterning cells into a medium-filled culture dish at high resolution. The advantages of the inkjet-based cell printing include: the lack of requirement for surface engineering, its chemical-free nature, and the lack of requirements for supporting hydrogels and the fabrication of masks, stamps or molds for each design pattern. We simply print multiple cell types suspended in a cell culture medium using two inkjet nozzles, and ink reservoirs are easily replaced to generate more complex cell patterns on a single surface. The first printed cell type does not need to be exposed to additional conditions to pattern the second cell type. We believe that the direct printing of mammalian cells into a medium is an unprecedented way to align cells in freeform patterns.

The basis of direct printing is the accumulation of cells at specific points on a substrate, which creates lines, solid areas or designed patterns. Each printed cell must be placed in an exact predetermined location on the medium-filled surface to ensure the precise mimicry of complex tissues. We fulfilled these requirements with a comprehensive study of printing processes. We first examined the rheological properties of cell suspensions, and carefully determined printing parameters, such as the actuation waveform, ejection frequency and back pressure of an ink reservoir, for stable and reproducible jetting. The high-speed stroboscopic imaging technique allowed us to observe the evolution of a jet formed by a cell-laden ink. The use of an appropriate volume of medium in a culture dish is also a critical factor in achieving high cell placement accuracy. A large volume increases the positional error of cells on the moving stage due to lapping waves, whereas a small volume decreases cell viability as it quickly dries during printing. High-speed cinematography allowed us to directly observe the complete trajectory of a cell printed into a medium from the moment of impact to anchoring onto the surface. These fundamental studies of the cell printing process with two different high-speed imaging techniques allowed us to achieve high-resolution printing on a liquid surface with positional deviation of ± 66 μm (1 sigma).

The fidelity and versatility of inkjet cell printing into a liquid medium are exemplified well in the printed patterns shown in Fig. 4 and 5. Cells were aligned into patterns and diverse local density gradients while the edges of the printed planes remained sharp. Local density gradient patterns were obtained using the drop-on-demand jetting function of inkjet printing. Without any surface modification or mold manufacturing, such patterns were fabricated with simple freeform inkjet printing in one step. More cell types can be printed without further complicated steps. Furthermore, printed cell migration assay models would provide reproducible and standardized gaps to allow researchers designing heterotypic cell migration assay models to utilize unlimited cell types and quantify their migration. Additionally, printed cell viability was closely analyzed immediately after printing for 7 days and for up to 8 passages (Fig. 3). Printed cell groups followed the proliferation curve of the control group, although the dish was not confluent after 7 days. The cell viability observed after printing partially verifies the safety of using a piezoelectric inkjet printer. Additional in-depth studies of the molecular impacts of printing are in progress.

As a case study, we applied direct cell printing to monitor the effect of geometric aspects of cellular populations on the effectiveness of viral infection. Unlike the homogeneous cells used in routine cell culture systems *in vitro*, living organisms contain heterogeneous cell types and cell geometries. The importance and correlation between cellular activity, such as virus infection, and heterotypic patterns comprising the population has been studied^40^. However, little is known about the effects of spatial complexity between heterogeneous groups, as these effects are difficult to study using conventional cell culture models. We created variably sized checkerboard patterns of HeLa and A549 cells in a medium-filled culture dish to examine the effect of population geometry on viral infection. Remarkably, the total amount of viral RNA in infected cells increased with the size of the inner squares, but not in A549 cell-only and HeLa cell-only patterns. These results indicated that variability in cell-to-cell interactions generated by the heterogeneous geometric patterning also influence on the population behavior, like viral infectivity. The underlying cause of this heterogeneity remain unclear.

In conclusion, the direct-printing approach developed here generated freeform patterns of multiple types of cells in a liquid environment. The controlled spatial locations of living cells were created simply by delivering viable cells suspended in a vial to a medium-filled culture dish through a drop-on-demand inkjet nozzle without inducing damage. This method may offer new opportunities to more accurately model critical cell-cell and tissue-tissue interfaces observed in living organisms. Furthermore, this technique may be applied for high-throughput analyses and drug screens.

## Methods

### Cell ink preparation

Cell ink was prepared by suspending cells in Dulbecco’s Modified Eagle’s Medium (DMEM, HyClone Laboratories Inc., Logan, Utah, USA) supplemented with 10% fetal bovine serum (FBS, HyClone Laboratories Inc., Logan, Utah, USA) and a 1% antibiotic/antimycotic solution (Sigma-Aldrich Corp., St. Louis, Missouri, USA). Mouse NIH3T3 fibroblasts and HEK293A human embryonic kidney cells were provided by Prof. Kyungtae Kim, POSTECH, Korea. Cells were trypsinized from a T-75 cell culture flask by adding 2 mL of 1x trypsin/EDTA (0.25%, 0.2 g L^−1^ EDTA) (HyClone Laboratories Inc., Logan, Utah, USA) for 3 min. DMEM (8 mL) was added to neutralize the trypsin/EDTA activity. The cell suspension was centrifuged at 300 × g for 2 min. The supernatant was removed by aspiration and the cell pellet was re-suspended in DMEM. The re-suspended cells were filtered through a 40-µm cell strainer. Filtered cells were counted using a hemocytometer to produce a cell concentration of 6 × 10^6^ cells mL^−1^.

The dynamic viscosity of the ink was measured using a rotational rheometer (Discovery HR-2, TA instruments Inc., Newcastle, Delaware, USA) with a cone and a plate (Al cone; 20 mm in diameter; 2° cone opening angle; 56-µm gap). An ink volume of 0.1 mL was loaded between gaps. Shear rates between 1 s^−1^ and 3,000 s^−1^ were applied to each ink for 60 seconds and the average time was monitored for the last 30 seconds. Temperature was precisely maintained at 25 °C. Measurements were repeated three times for each condition (*n* = 3).

### High-speed imaging of cell-laden drop jetting, impacting and sinking

We employed two imaging techniques to observe the movement of cells in ambient air and sinking in a liquid-filled bath. First, the evolution of jet morphology was observed using a CCD camera (avA1000-100gm, Basler AG Inc., Ahrensburg, Germany), zoom lens, and nano-pulsed light (150 ns, NP-1A, Sugawara Laboratories Inc., Kawasaki, Japan) based on stroboscopic flash photography to design the proper characteristics of cell-laden drops, such as the velocity and number of satellite drops. Each stroboscopic image was recorded at 10 frames per second by synchronizing this equipment using a NI DAQ boards (NI USB6351, National Instruments Corp., Austin, Texas, USA), and the velocity of the leading drop was evaluated. Next, cell behaviors were observed at 12,000 frames per second using a high-speed camera (Fastcam SA3 120 K M3, Photron Inc., San Diego, California, USA), zoom lens, and halogen light (LS-F150HS Light Bank, Seokwang Optical Co., Ltd, Hwaseong, Korea) to analyze the dynamics of cell impact and sinking. The cell-laden drop impacted onto the surface of the transparent acrylic bath filled with a water-miscible fluid, such as ethanol. After the drop impacted the DMEM-filled bath, sinking cells were tracked and the sinking displacement was measured. The measurement was repeated 10 times.

### Printing system set-up

The printing system was a piezoelectric inkjet printer system (Jetlab®II, MicroFab Inc., Plano, Texas, USA) with an x-y moving stage and z-axis elevating nozzle. An inkjet nozzle composed of an 80-µm diameter glass capillary (MJ ATP 01 80, Microfab Inc., Plano, Texas, USA) was used. The printer’s moving speed (*v*, 5 ≤ *v ≤ *80 mm s^−1^) and acceleration (*a*, 20 ≤ *a* ≤ 320 mm s^−2^) were controlled. Jet formation is influenced by the piezoelectric actuator, which is controlled by the electrical waveforms. The applied voltage was set to ± 70 V to generate a bipolar waveform. The jetting frequency was set to ~200 Hz to generate a stable and clear jet. A tailor-made nozzle adaptor was installed to reduce the stand-off distance (*d*), which is the height between the tip of an inkjet nozzle and the substrate. The selected range of the stand-off distance was 1.0 ≤ d ≤ 20.0 mm. Additionally, the printer was equipped with a UV-C lamp for sterilization before and after printing. Ethanol was filtered through a 0.45-µm pore nylon filter (GE Whatman Inc., Buckinghamshire, UK) and used as the cleaning solution. The temperature and humidity of the printing chamber were constantly measured and controlled at 25 °C and 50%, respectively, using sterilized humidifiers during cell printing to avoid substrate dehydration.

### Substrate preparation

Substrates were vacuum-gas plasma-treated 10-cm cell culture dishes (PRIMARIA®, Corning Inc., Corning, New York, USA) and coated with a 0.01% poly-l-lysine solution (PLL, Sigma-Aldrich Corp., St. Louis, Missouri, USA). Substrates were treated with PLL to reduce cell adhesion time. Approximately 3–4 mL of DMEM was added to reduce cell death caused by dehydration and the impact force. Bare plastic cell culture dishes without medium were prepared to determine the resolution of the original inkjet cell printing conditions on solid and not liquid substrates.

### Data analysis

The resolution of various printing conditions was quantified by analyzing images of printed cells. One hundred dots of single drops were printed on 10 × 10 arrays with a drop spacing of 2 mm on both the x and y axes. A CCD camera linked to the printing system with an x-y moving stage was controlled with a manually designed script and automatically recorded images of the results. MATLAB software (Mathworks Inc., Natick, Massachusetts, USA) extracted and converted the locations of each cell in the images to coordinates. The originally desired printing point was estimated by overlaying and averaging the coordinates of all cells from the 100 printed dots. The distances between cell coordinates and the actual printing point were then calculated. Printing resolution values obtained for each condition were compared using the root-mean-square deviation of the x and y distances. For in line printing, lines with drop spacing values ranging from 20 to 100 µm were printed. The predetermined lengths of each complete line were 2 cm. A series of images was captured with the stage moving horizontally in 60-µm increments at each time point. Line thickness was measured from line images. The number of cells in a single drop was extracted using MATLAB software. One thousand five hundred drops were analyzed to estimate the average number of cells.

### Cell pattern design and printing

Cell patterns were printed from monochrome bitmap images. Multicolor images were scaled to printable pixels by considering the total image size and drop spacing and were divided into red, green, and blue files. Images with equivalent cell densities were overprinted 5 times with a constant drop spacing. For images of locally divergent cell densities such as the RGB color chart and “The Eiffel Tower”, files of each color were again divided into 5 separate files to depict intensity gradients over 5 levels. The desired cell density was controlled by adjusting the drop spacing between 80 and 140 µm, but the drop spacing was fixed throughout the printing process for a single image once it was established.

### Cell viability tests

Post-printing cell viability was immediately measured using a LIVE/DEAD® Viability/Cytotoxicity kit for mammalian cells (Invitrogen Corp., Carlsbad, California, USA). Cells used for these assays were printed in 10-cm cell culture dishes filled with 3 mL of DMEM for consistency with the original patterning conditions. Control groups were prepared by manual pipetting cells. Cells from both control and printed groups were treated with the LIVE/DEAD® assay reagent for 30 min according to the manufacturer’s instructions. The numbers of green live cells and red dead cells were automatically counted using an automatic cell counter (Countess II FL Automated Cell Counter, Invitrogen Corp., Carlsbad, California, USA). Cell proliferation was also assessed in both control and printed groups using Cell Counting Kit 8 (CCK 8, Dojindo Laboratories Co., Ltd, Kumamoto, Japan). The assay reagent was added to the cells and incubated for 2 hours. UV absorbance was measured at 450 nm for each printed cell pattern on days 1, 3, 5, and 7 using a spectrophotometer (SPECTROstar Nano, BMG Labtech Inc., Ortenberg, Germany). Images of cells stained with blue Hoechst 33342 dye were captured on days 1, 7, 14, and 21 using a fluorescence microscope (Ti-s, Nikon Corp., Tokyo, Japan) to further assess the long-term viability and morphology of cells grown in maintenance cultures.

### Imaging printed cell patterns

Cells were labeled with fluorescent dyes to visualize patterns with multiple cell types. Green (CellTrace™ CFSE Cell Proliferation Kit, Invitrogen Corp., Carlsbad, California, USA), red (CellTracker™ Red CMTPX, Invitrogen Corp., Carlsbad, California, USA), and blue (CellTracker™ Blue CMAC Dye, Invitrogen Corp., Carlsbad, California, USA) fluorescent dyes were used. Suspension cells were stained for 30 min, filtered through a 40-µm cell strainer and counted to generate fluorescent bio-inks containing 6 × 10^6^ cells mL^−1^. Images of the fluorescent printed cell patterns were captured using a fluorescence microscope (Ti-s, Nikon Corp., Tokyo, Japan). A magnification of 20x was primarily used to image large areas of the patterns.

### Influenza virus infection and measurements of the intracellular viral RNA concentration

The influenza A virus (PR8 strain) was obtained from Dr. Adolfo Garcia-Sastre, Mount Sinai School of Medicine. Ten units of the median tissue culture infectious dose (TCID50 mL^−1^) of Influenza A virus (PR8) were added to the printed A549 and HeLa cells for 3 hours in serum-free DMEM. Cells were then washed with PBS, followed by further incubations in DMEM (10% FBS) for the indicated times. After the incubation, cells were washed with PBS, harvested in RNAiso plus (TAKARA Bio Inc., Shiga, Japan) and the intracellular RNA was extracted according to the manufacturer’s instructions. The purity and quantity of total RNAs from each sample were determined by measuring the absorbance at 260 and 280 nm. RNA from each sample (1 µg) was reverse transcribed with random hexamers using the ImProm-II Reverse transcriptase system (Promega Corp., Madison, Wisconsin, USA) according to the manufacturer’s protocol. Quantitative real-time PCR of cDNA samples was performed with the StepOne Plus Real-Time PCR system (Applied Biosystems Inc., Foster City, California, USA). The viral genomes were detected by measuring influenza HA expression using a pair of oligomer (5-TTGCTAAAACCCGGAGACAC-3, 5-CCTGACGTATTTTGGGCACT-3). The levels of the viral genome were normalized to the levels of the host GAPDH mRNA using a pair of oligomer (5-aggtgaaggtcggagtcaac-3, 5- tggaagatggtgatgggatt-3).

### Statistical analysis

The average distance of cells from the center and line width was analyzed using 95% confidence intervals (CIs). Cell proliferation values are expressed as means, with error bars representing the ± standard deviations (s.d.). The number of cells measured in experiments with varying drop spacing distances and the virus infection analysis are expressed as means, with error bars representing the ± standard errors (s.e.). The actual and calculated numbers of printed cells were compared using two-way ANOVA followed by Tukey’s test with OriginPro 2016 software (OriginLab Corp., Northampton, Massachusetts, USA). Two-way ANOVA was also applied to determine the differences in the cell proliferation rate between the printed and control groups.

## Electronic supplementary material

Supplementary information video

Supplementary information pdf
