# Publisher Correction: Freeform micropatterning of living cells into cell culture medium using direct inkjet printing

**DOI:** 10.1038/s41598-018-20338-9

**Published:** 2018-01-23

**Authors:** Ju An Park, Sejeong Yoon, Jimin Kwon, Hesung Now, Young Kwon Kim, Woo-Jong Kim, Joo-Yeon Yoo, Sungjune Jung

**Affiliations:** 1 Department of Creative IT Engineering, Pohang University of Science and Technology (POSTECH), 77 Cheongam ro, Nam-gu, Pohang Kyungbuk 37673 Republic of Korea; 2 School of Interdisciplinary Bioscience and Bioengineering, Pohang University of Science and Technology (POSTECH), 77 Cheongam ro, Nam-gu, Pohang Kyungbuk 37673 Republic of Korea; 3 Department of Life Sciences, Pohang University of Science and Technology (POSTECH), 77 Cheongam ro, Nam-gu, Pohang Kyungbuk 37673 Republic of Korea; 4 Department of Mechanical Engineering, Pohang University of Science and Technology (POSTECH), 77 Cheongam ro, Nam-gu, Pohang Kyungbuk 37673 Republic of Korea

Correction to: *Scientific Reports* 10.1038/s41598-017-14726-w, published online 03 November 2017

The original HTML version of this Article contained a typographical error in the publication date ‘03 November 2017’ which was incorrectly given as ‘06 November 2017’. This has now been corrected in the HTML version of the Article.

