# Author Correction: Freeform micropatterning of living cells into cell culture medium using direct inkjet printing

**DOI:** 10.1038/s41598-020-70348-9

**Published:** 2020-08-04

**Authors:** Ju An Park, Sejeong Yoon, Jimin Kwon, Hesung Now, Young Kwon Kim, Woo-Jong Kim, Joo-Yeon Yoo, Sungjune Jung

**Affiliations:** 1 Department of Creative IT Engineering, Pohang University of Science and Technology (POSTECH), 77 Cheongam ro, Nam-gu, Pohang, Kyungbuk 37673 Republic of Korea; 2 School of Interdisciplinary Bioscience and Bioengineering, Pohang University of Science and Technology (POSTECH), 77 Cheongam ro, Nam-gu, Pohang, Kyungbuk 37673 Republic of Korea; 3 Department of Life Sciences, Pohang University of Science and Technology (POSTECH), 77 Cheongam ro, Nam-gu, Pohang, Kyungbuk 37673 Republic of Korea; 4 Department of Mechanical Engineering, Pohang University of Science and Technology (POSTECH), 77 Cheongam ro, Nam-gu, Pohang, Kyungbuk 37673 Republic of Korea

Correction to: *Scientific Reports*https://doi.org/10.1038/s41598-017-14726-w, published online 03 November 2017

In Figure 6b, data from MEF cells were mistakenly inserted in place of data from A549/HeLa cells. The correct Figure 6b appears as Figure 1. The conclusions are not affected by this change.

**Figure 1:**
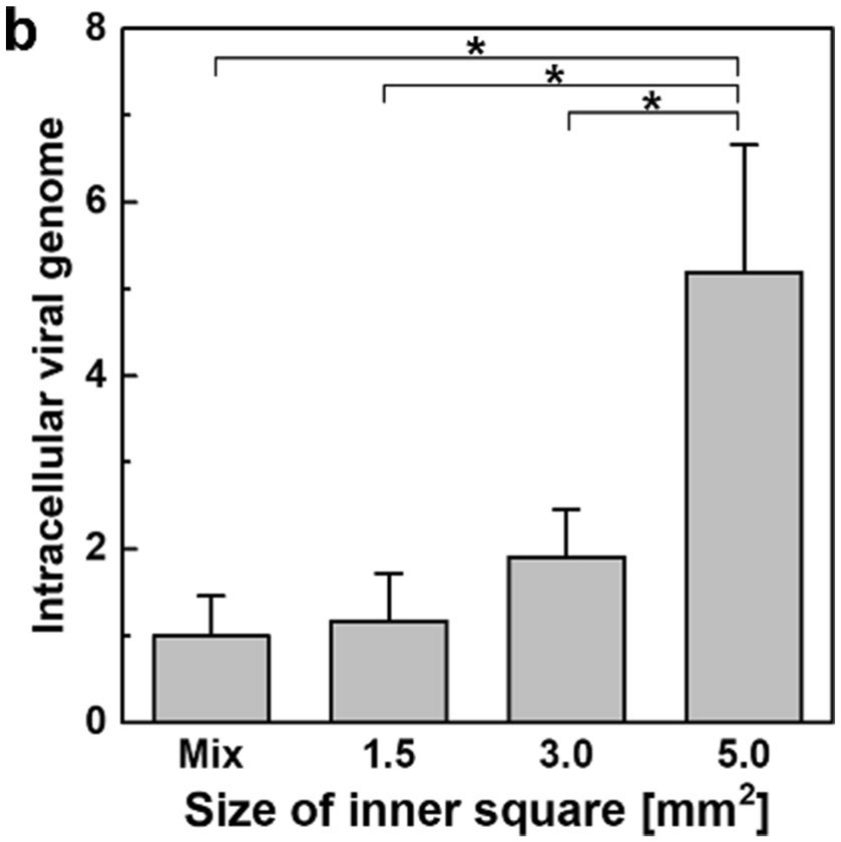
(**b**) After patterning heterotypic A549 and HeLa cells according to the designs shown in (**a**), cells were incubated for 48 h and infected with influenza virus (10 TCID50 mL^−1^) for 24 h. Cells were harvested and the amount of the influenza HA viral RNA (normalized to GAPDH levels) was measured using quantitative real-time PCR. Means and standard errors were obtained from three independent experiments and analyzed by one-way ANOVA followed by the Newman–Keuls post hoc test, *p < 0.05.

