# Supplementary material for: Freeform micropatterning of living cells into cell culture medium using direct inkjet printing
Source: Sci Rep. 2017 Nov 3;7:14610. doi: 10.1038/s41598-017-14726-w (PMC5668285; doi:10.1038/s41598-017-14726-w)
Supplement: Supplementary file 2 — Supplementary information pdf [file 41598_2017_14726_MOESM2_ESM.pdf]

Freeform micropatterning of living cells into cell culture medium using direct inkjet printing

Ju An Park[1], Sejeong Yoon[2], Jimin Kwon[1], Hesung Now[3], Young Kwon Kim[4], Woo-Jong Kim[3], Joo-Yeon Yoo[3] and

Sungjune Jung[1,2]*.

[1] Department of Creative IT Engineering, Pohang University of Science and Technology (POSTECH), 77 Cheongam ro, Nam-gu, Pohang, Kyungbuk 37673, Republic of Korea.

[2] School of Interdisciplinary Bioscience and Bioengineering, Pohang University of Science and Technology (POSTECH), 77 Cheongam ro, Nam-gu, Pohang, Kyungbuk 37673, Republic of Korea.

[3]Department of Life Sciences, Pohang University of Science and Technology (POSTECH), 77 Cheongam ro, Nam-gu, Pohang, Kyungbuk 37673, Republic of Korea.

[4] Department of Mechanical Engineering, Pohang University of Science and Technology (POSTECH), 77 Cheongam ro, Nam-gu, Pohang, Kyungbuk 37673, Republic of Korea.

* Emails: sjjung@postech.ac.kr

Supplementary information

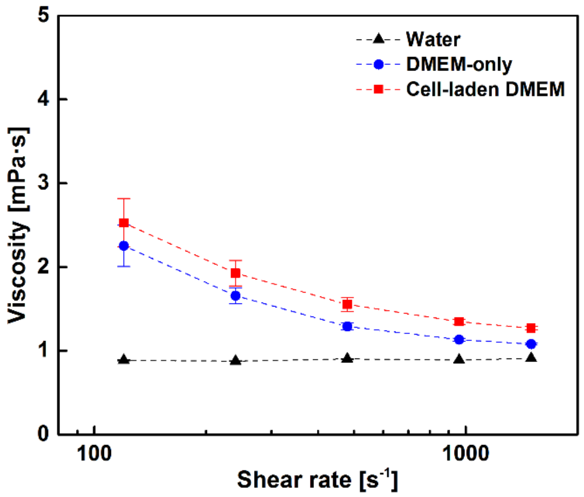

Supplementary Figure 1. Shear viscosity of the ink. The viscosities of the cell-laden ink and its base fluid, DMEM, were measured and compared with that of a Newtonian fluid, water. Cell-laden ink had a basal viscosity of less than 20 mPa·s and exhibited a decrease in the shear-thinning viscosity as the shear rate increased. The detailed investigation on the effects of fluid properties on printing a cell-laden ink can be found in our previous study[37].

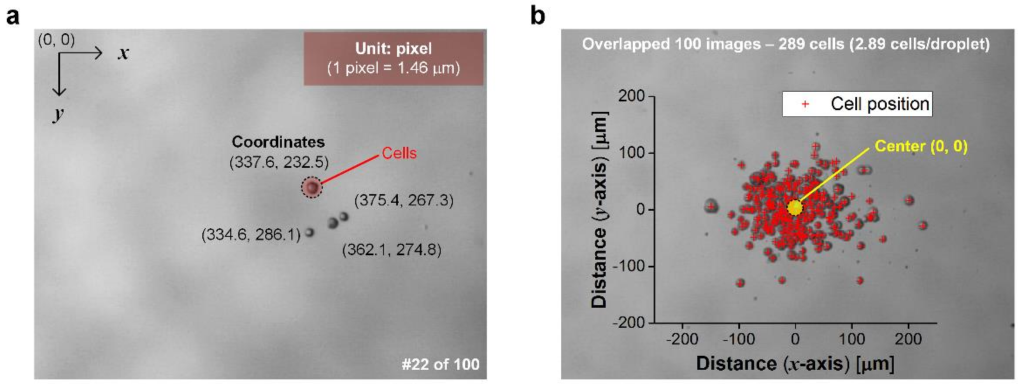

Supplementary Figure 2. Cell coordinate extraction process. (a) An image of cells in one printed droplet. Images were taken with printer-mounted CCD camera right after cell positioning. Position of deposited cells were obtained by extracting coordinates from MATLAB image processing steps. (b) Overlapped images of 100 printed droplets. Center positon was obtained by averaging distribution of cell coordinates. Weighted sums were applied to closely bound neighbor cells.

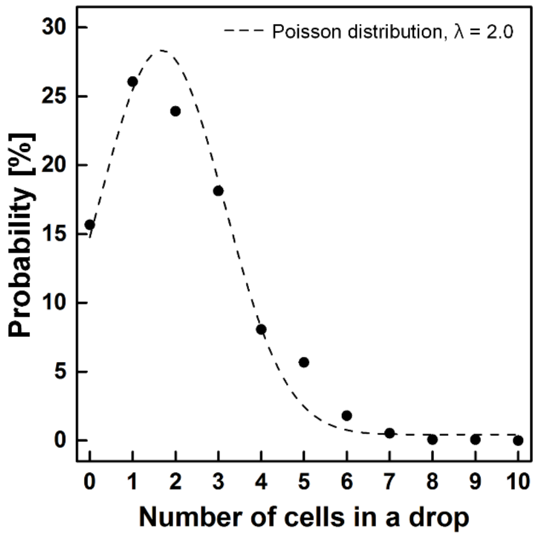

Supplementary Figure 3. Number of cells in printed drops. The distribution of the number of cells printed in 1,500 drops with a density of 6×10^6 NIH3T3 cells mL^{-1} is shown. The dashed line indicates the Poisson distribution, $\lambda = 2.0$.

Supplementary Video. A movie based on high-speed cinematographic images of sinking cells after a drop impacting (Fig. 1D). Images were obtained at a rate of 12,000 frames per second.
